# Spontaneous Ureterocolic Fistula between Nonfunctioning Kidney Transplant Ureter and Colon in Setting of Diverticulitis

**DOI:** 10.1155/2021/5572067

**Published:** 2021-07-20

**Authors:** Madison K. Krischak, Jeffrey R. Ord, Ashton A. Connor, Andrew S. Barbas

**Affiliations:** ^1^Duke University School of Medicine, 2608 Erwin Rd, Suite 210, Durham, NC 27705, USA; ^2^Division of Abdominal Transplant Surgery, Department of Surgery, Duke University School of Medicine, 2301 Erwin Rd, Durham, NC 27705, USA

## Abstract

Ureterocolic fistula is a rare condition that most commonly occurs in the setting of diverticular disease. The development of a ureterocolic fistula following kidney transplantation is even rarer, with no prior cases in the literature to our knowledge. We describe the case of a patient with three prior failed kidney transplants who developed a fistula between the sigmoid colon and nonfunctioning renal transplant ureter in the setting of diverticulitis.

## 1. Introduction

The formation of fistulas in the setting of a diverticular disease is relatively rare, occurring in only 1% of cases [1]. Fistula formation with the ureter is particularly rare, with only 11 previous cases reported in the literature [2]. Ureterocolic fistulas most commonly affect the left ureter given its proximity to the sigmoid colon. Presentation, diagnosis, and management of this condition vary according to the functional status of the affected upper urinary tract. In patients with functional kidneys, the recommended treatment is resection of the affected colon without surgical intervention of the urinary system. However, in the setting of a nonfunctioning kidney, resection of the affected urinary system is indicated [3]. We present a case of ureterocolic fistula in the setting of nonfunctional renal transplant graft with subsequent colonic resection and transplant ureteronephrectomy. To our knowledge, this is the first published example of a ureterocolic fistula with a nonfunctional renal transplant ureter in the setting of diverticulitis.

## 2. Case Presentation

The patient is a 57-year-old female with type 1 diabetes complicated by end-stage renal disease. The patient had an extensive transplant history including a kidney transplant in 1996, followed by a simultaneous kidney and pancreas transplant in 2000, followed by another kidney transplant in 2009. All three kidneys failed due to chronic rejection. The patient had returned to hemodialysis and was anuric at baseline. Her pancreatic graft remained functional, and she was continued on immunosuppression with 0.5 mg tacrolimus and 5 mg prednisone daily. She underwent transplant nephrectomy of the most superficial nonfunctional renal graft in 2017 due to its enlarging size and discomfort. The patient's other significant medical history included hepatitis C, history of pneumocystis pneumonia, chronic anemia, severe peripheral artery disease, and left lower extremity nonhealing wound status post amputation.

**Figure 1:**
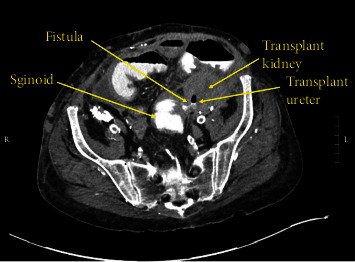
Axial CT of the abdomen and pelvis with rectal contrast demonstrating fistulous connection between the sigmoid colon and left renal transplant ureter.

**Figure 2:**
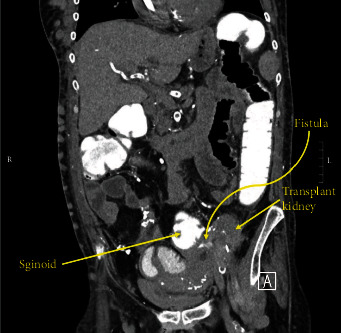
Sagittal CT of the abdomen and pelvis with rectal contrast demonstrating fistulous connection between the sigmoid colon and left renal transplant ureter.

The patient presented to our institution's emergency department with abdominal pain and low-grade fevers for 48 hours. At presentation, her vital signs were stable and she was nontoxic appearing with localized tenderness in the left lower quadrant. Initial computed tomography (CT) imaging of the abdomen and pelvis demonstrated a fistulous connection between the sigmoid colon and the patient's left nonfunctioning kidney transplant ureter (Figures 1 and 2). The patient was started on intravenous piperacillin-tazobactam for broad antibiotic coverage. Flexible sigmoidoscopy and biopsy were completed and were negative for evidence of malignancy as a cause of fistula. She was taken to the operating room for sigmoid colectomy with colostomy and transplant ureteronephrectomy.

In the operating room, the fistula was identified. The affected colon was mobilized, proximally and distally, and removed. Following colonic resection, the transplant ureter was identified, ligated, and divided near the bladder. The kidney graft was mobilized and removed. Hemostasis was achieved in the pelvis, but a laceration of the spleen was identified, and splenectomy was required.

Once hemostasis was achieved, the colorectal team formed an end colostomy. At this point in the case, the patient had significant hypotension requiring placement of a central line, increasing vasopressor support, and blood products. At the end of the case, she was transported to the intensive care unit (ICU) hemodynamically unstable and intubated. Operating room pathology demonstrated diverticulosis with perforation and explanted kidney with extensive necrosis and pyonephrosis. Given intraoperative findings, blood cultures were obtained and the patient was continued on piperacillin-tazobactam with addition of vancomycin and micafungin at recommendation of infectious disease colleagues.

Postoperatively, the patient required vasopressor support and ICU level care. She returned to hemodialysis on post-op day (POD) 1 and was weaned off vasopressors on POD 2. Blood cultures remained negative at 48 hours; thus, antimicrobials and antifungals were discontinued. The patient's recovery was otherwise uneventful. She was deemed medically fit for discharge on POD 10.

Two weeks following discharge, the patient represented in septic shock and was found to have diffuse purulent peritonitis upon exploratory laparotomy. Her subsequent hospital stay was prolonged and complicated, although she was eventually discharged to a skilled nursing facility. Unfortunately, she passed away shortly thereafter during a subsequent admission for osteomyelitis.

## 3. Discussion

Fistula formation in the setting of diverticulitis is rare, occurring in only 1% of cases [1]. The most common fistulas formed are colovesical and colovaginal [4], with ureterocolic fistulas being extremely rare in the literature. Historically, ureterocolic fistulas have formed in the setting of tuberculosis. Other notable causes include ureterolithiasis, pyelonephritis, prior surgical intervention, trauma, Crohn's disease, diverticulitis, radiation therapy, and malignancy [5]. Ureterocolic fistula due to diverticulitis is exceedingly rare, with only 11 prior cases reported in the literature to our knowledge [2]. Of those cases, none have taken place in patients with nonfunctioning kidney transplant ureters.

Diagnosis of ureterocolic fistula is dependent on the functional status of the affected urinary system. Importantly, most ureterocolic fistulas will present with both urinary symptoms such as urinary tract infection, pneumaturia, and fecaluria, and abdominal pain [2, 6]; however, in the setting of a nonfunctional transplant ureter and kidney, urinary symptoms are less likely. Thus, awareness of this complication in absence of characteristic symptoms is key when considering the differential for abdominal pain in this population. Diagnosis is typically made via retrograde urethrography, excretory urography (showing contrast material entering the bowel), or barium enema (with contrast media entering the ureter) [5]. However, in the setting of a nonfunctional transplant kidney these tests are of limited value and diagnosis more heavily relies on CT imaging. When a ureterocolic fistula is suspected, appropriate imaging studies are essential for quick diagnosis and prevention of urosepsis.

Similar to diagnosis, surgical management of ureterocolic fistula is also dependent on the functional status of the kidney. In cases of functional kidneys, it is recommended that the affected bowel be resected without intervention on the kidney or ureter [3, 5]. In more severe cases, however, the decision may be made to dissect the fistula and reanastomose the ureter [5]. Some rare cases of conservative management with antibiotics have been reported [7], but this is generally not recommended over surgical management. In the setting of nonfunctioning kidney as in this case, the recommended management includes both resection of the affected bowel and ureteronephrectomy to decrease risk for urosepsis in the nonfunctioning upper urinary tract [6, 8].

In this case, surgical management of the patient's ureterocolic fistula was successful, given the patient stabilized and achieved adequate ostomy output, ambulation, and pain control with oral medications prior to discharge home. Unfortunately, the patient represented in septic shock two weeks later; however, her presentation is thought to be predominantly a consequence of compromised immune function given her immunosuppressive medications and recent splenectomy. Importantly, asplenic patients are at heightened risk of overwhelming postsplenectomy infection (OPSI) [9]. Although this patient received appropriate antibiotics during her immediate postoperative period, some recommendations suggest that asplenic individuals with particularly high risk of infection, including those with solid organ transplants, should receive daily antibiotic prophylaxis for the duration of their immunosuppression in addition to appropriate vaccines; however, the data is limited and these recommendations vary [10]. While this patient's surgical management of ureterocolic fistula was successful, her postoperative course was significantly impacted by her heightened immunocompromised state and should be a consideration in all asplenic patients with surgically managed ureterocolic fistula.

Discussion of ureterocolic fistula management in the renal transplant population is increasingly important. If caught early in a patient with a functional graft, the graft may be spared and urosepsis avoided. However, early detection of this phenomenon can be difficult in the anuric patient with failed transplant grafts due to lack of symptoms and thus relies heavily on imaging. As the population with renal transplants expands and ages, they face increased risk of diverticular disease. As such, it can be postulated that although rare, the incidence of ureterocolic fistulas in the transplant population may increase over time. Awareness of this complication and application of the appropriate diagnostic tools and surgical management based on the functional status of the renal transplant are crucial.

## Data Availability

No data were used to support this study.
